# The Foreign Journals: Animal Chemistry

**Published:** 1838-10

**Authors:** 


### ANIMAL CHEMISTRY.

### *On the Chemical Composition of Human Lymph.* By R. T. Marchand and C. Colberg, of Halle.

It is very difficult to obtain a sufficient quantity of pure human lymph to subject to satisfactory examination: and, when it is remembered how great are the differences of opinion respecting the composition of the blood, although this fluid may be obtained in large quantities, and may be examined in the body itself, the difficulties attending a chemical examination of lymph may well be appreciated: and yet a correct chemical analysis of the composition of lymph, as compared with that of the blood, is a subject of considerable importance; for, by this means, the quantity of lymph contained in the blood may be estimated; since the afflux of lymph to the blood, and its existence in that fluid in an unchanged state, (at least the lymph granules,) is a fact which has been satisfactorily proved.

The examination of lymph was conducted by each of the above experimenters, independently of the other. The lymph which was selected for experiment was taken from a wound on the back of the foot, which very obstinately resisted the healing process. The quantity thus obtained was but small; its specific gravity was 1.037. After having been set apart for some time, there collected at the bottom of the vessel a cobwebby coagulum of fibrin, which, when filtered, dissolved in ether, and then dried in a water bath, amounted to 0.52 per cent. The fluid which remained, of a somewhat opaline appearance, and of a light yellow colour, was about the consistence of almond oil. When mixed with thirty parts of water, the appearance was still slightly opaline, but there was no deposit after long standing. Alcohol and bichloride of mercury immediately precipitated tender, white flocculi. The fluid had a strongly alkaline reaction, restoring the reddened litmus; a circumstance of interest, and probably serving to explain the strongly alkaline reaction which is found in purulent matter upon fresh secreting surfaces, which quality ceases in pus taken from these surfaces, which is neutral. When warmed in the water bath to about 204° Fahr., the lymph coagulated completely, from the presence of albumen. Heated to 212° F., and allowed to remain for some time at this temperature, it became a hard grey mass, which could be easily powdered. 6.798 grains left a residuum of 0.209 grains of solid constitutents, = 3.074 per cent. When this residuum was treated with ether, it suffered a loss of 0.018 grains; and, when the ether was evaporated from a watch-glass by the gentlest heat, there remained reddish coloured globules of fatty matter, which, when dissolved in alcohol, and examined by a powerful microscope, appeared to be converted into an oily and crystalline substance. By increasing the temperature, this fatty matter was evaporated; the vapour possessing a very disagreeable smell, and producing considerable irritation of the eyes. The material which had been exhausted by ether was next treated with boiling water, leaving a residuum of 0.065 grains, consisting of albumen and fibrin. The filtered fluid was quite blue; and, after evaporation in a water bath, there remained a whitish yellow, saltish residuum, which, evaporated to dryness, showed a feeble alkaline reaction. Heated to a red heat, there remained a residuum of 0.105 grains, which effervesced with acids, and possessed a much more evident alkalinity; probably owing to decomposition of a lactate of soda, although the existence of a carbonate of an alkali was very probable. In the solution of the residuum, there was produced by nitrate of silver a white precipitate, insoluble in nitric acid, soluble in ammonia. A few drops of the chloride of platinum threw down a slight precipitate, which had increased in quantity on the following day. Chloride of barium caused a copious precipitate, which was partially soluble in hydrochloric acid. In the solution when filtered, and which was quite clear, caustic ammonia again caused instantaneously a copious turbidity. Another part of the evaporated lymph was, as above mentioned, treated with ether, with water, and, lastly, with alcohol. The residuum gave rise to a strong, dark yellow coloration in the outer part of the flame of a blowpipe. In the alcoholic solution, tincture of galls produced a brownish yellow, flocculent precipitate, (osmazome.) The remains of that portion which had been treated with alcohol, water, and ether, and the residue of the alcoholic solution, were evaporated to dryness, heated red hot, and reduced to an ash. This was dissolved in water, which took up all but a few trifling grey flocculi, which, dissolved in a small quantity of hydrochloric and nitric acids, produced a blue precipitate with a solution of the ferrocyanate of potass. This, too, was very marked in the aqueous solution. Oxalate of ammonia produced a precipitate, having the characteristics of oxalate of lime; and, in the residuum of the filtered fluid, the blowpipe proved the existence of soda and potass.

From what has been said, the following may be inferred to be the constituents of human lymph:

| | |
|---|---|
| Water | 96,926 |
| Fibrin | 0,520 |
| Albumen | 0,434 |
| Osmazome (and loss) | 0,312 |
| Fatty oil<br>Crystalline fat | 0,264 |
| Chloride of sodium<br>Chloride of potassium<br>Carbonate and lactate of an alkali<br>Sulphate of lime<br>Phosphate of lime and oxide of iron | 1,544 |
| | 100,000 |

From the above-mentioned experiments, as well as from analyses of lymph both of man and animals by other chemists, it is evident that there exists a very near resemblance between the chemical composition of lymph and of blood. In both there are nearly the same salts; in both are there albumen, fibrin, osmazome, and probably a very similar fat. The proportion of albumen in the blood is, indeed, considerably greater than in lymph; instead of which there appears to be here a much larger quantity of fibrin, but we cannot certainly say that this is identical with that of the blood. The colouring matter of the blood is also wanting in lymph. Meanwhile it cannot be denied that most of the above constituents form also a great part of the other animal fluids, if we except those which are excreted. Albumen and fat we may indeed always meet with, but not fibrine; although it is very probable that this has been overlooked when its presence has not been suspected, as may be concluded from the observations which have been made on fibrinous urine.

*Archiv für Anatomie, Physiologie, &c., von Dr. J. Müller.* No. 2. 1838.

---

*Microscopical Researches on the Composition of Vaccine Fluid.* By M. Dubois.

M. Dubois has found that the crystalline appearance found in vaccine lymph, and alluded to in our eleventh Number, is owing to the presence of hydrochlorate of ammonia: that these crystals form very rapidly, and with great beauty, when the vaccine virus is quite free from any organized matters, bits of membrane, globules of pus or of blood. These crystals were immediately dissipated by the action of heat. M. Pelletier also states that he is satisfied of the existence of hydrochlorate of ammonia in vaccine lymph.

*Bulletin de l'Academie de Médecine.* No. 16. 1838.

---

*Microscopical Examination of Pus, Mucus, and Effused Fluids.* By Dr. Mandt.

Beneath the microscope, pus appears to be globules swimming in a fluid. If the globules are separated from the fluid in which they swim, by filtration, the limpid portion which passes through (i. e. the serum,) presents no trace of globules when examined by the microscope. Its appearance is exactly that of albumen dissolved in water. When heated it immediately coagulates, &c. The separation of pus into globules and fluid often takes place spontaneously, when a very fluid pus is left to itself. Mucus and various effusions, the serum which is found in the cellular membrane and cavities in dropsy, &c., are formed in exactly the same manner. The globules which have not passed through the filter are of two kinds: one, commonly spoken of as globules of pus, mucus, saliva, &c., are larger; their diameter being about one-hundredth of a millimètre. When these globules are compared with those of coagulated fibrin, either in the clot or in false membranes, or in the fibrils which are formed by the fibrin of blood when it is agitated with some albumen which prevents the fibrin forming itself into more solid membranes; they are found to be all of the same size, the same appearance, the same form, and they exhibit the same phenomena with chemical agents. Effusions, whatever may have been the time of their continuance, exhibit the same globules. Dr. Mandt proposes to call these *fibrinous globules:* they owe their existence to the fibrin secreted from the blood, and coagulated external to the vessels. Hence it follows, that the presence of these fibrinous globules does not prove that the liquid in which they are found contains pus; and that any inference in favour of the existence of pus drawn from them might induce an error. The second kind of globules, (the diameter of which varies from one four-hundredth to one five-hundredth of a millimètre) which are found mixed with the globules of pus, belong to the globules of albumen coagulated by the salts of the serum. Their number is greater in proportion as the serum abounds in salts. They are sometimes found among the globules of fat of different diameters. It may therefore be inferred that pus and mucus do not differ in their main constituent parts; that water, albumen, and fibrinous globules chiefly constitute them, and that it is but the relative proportion of these parts and the salts which exist in these fluids, which can serve to establish a difference. All the experiments which have hitherto been made to ascertain the distinction between pus and mucus, have therefore been inconclusive. These secretions owe their origin to the principal constituents of the blood, by means of exosmose. They are all found in these fluids, excepting the proper globules of the blood (colouring matter) which are unable to escape from the pores of the blood-vessels, in consequence of their size. It is thus demonstrated that we lose continually by the secretions (the mucus, urine, saliva, &c.) a portion of the fibrine contained in the blood.

*Revue Médicale Française et Etrangère. December,* 1837.

